# Concentration of the Most-Cited Papers in the Scientific Literature: Analysis of Journal Ecosystems

**DOI:** 10.1371/journal.pone.0000005

**Published:** 2006-12-20

**Authors:** John P. A. Ioannidis

**Affiliations:** 1 Clinical and Molecular Epidemiology Unit, Department of Hygiene and Epidemiology, University of Ioannina School of Medicine Ioannina, Greece; 2 Biomedical Research Institute, Foundation for Research and Technology-Hellas Ioannina, Greece; 3 Institute for Clinical Research and Health Policy Studies, Tufts University School of Medicine Boston, Massachusetts, United States of America; South African National Bioinformatics Institute, South Africa

## Abstract

**Background:**

A minority of scientific journals publishes the majority of scientific papers and receives the majority of citations. The extent of concentration of the most influential articles is less well known.

**Methods/Principal Findings:**

The 100 most-cited papers in the last decade in each of 21 scientific fields were analyzed; fields were considered as ecosystems and their “species” (journal) diversity was evaluated. Only 9% of journals in Journal Citation Reports had published at least one such paper. Among this 9%, half of them had published only one such paper. The number of journals that had published a larger number of most-cited papers decreased exponentially according to a Lotka law. Except for three scientific fields, six journals accounted for 53 to 94 of the 100 most-cited papers in their field. With increasing average number of citations per paper (citation density) in a scientific field, concentration of the most-cited papers in a few journals became even more prominent (p<0.001). Concentration was unrelated to the number of papers published or number of journals available in a scientific field. Multidisciplinary journals accounted for 24% of all most-cited papers, with large variability across fields. The concentration of most-cited papers in multidisciplinary journals was most prominent in fields with high citation density (correlation coefficient 0.70, p<0.001). Multidisciplinary journals had published fewer than eight of the 100 most-cited papers in eight scientific fields (none in two fields). Journals concentrating most-cited original articles often differed from those concentrating most-cited reviews. The concentration of the most-influential papers was stronger than the already prominent concentration of papers published and citations received.

**Conclusions:**

Despite a plethora of available journals, the most influential papers are extremely concentrated in few journals, especially in fields with high citation density. Existing multidisciplinary journals publish selectively most-cited papers from fields with high citation density.

## Introduction

Despite a very large number of scientific journals (probably exceeding 100,000 worldwide), the concentration of scientific information is skewed to a minority of journals that publish the majority of the articles (Bradford's law) and receive the majority of the citations. In the Web of Knowledge, a core of 2,000 scientific journals publishes about 85% of all articles and 95% of cited articles [1], [2]. Furthermore, the citation impact of scientific articles is highly uneven, even within the same journal, following a log-normal distribution where 20% of articles account for 80% citations (the “20/80 law”) [3]. It would be interesting to examine the diversity of journals that are responsible for publishing the most-cited articles in each scientific field. Several questions may be posed. What is the range of this diversity? Does it differ across scientific disciplines? What is the relative role of multidisciplinary journals? Do reviews differ from original articles in their concentration?

To answer these questions, I considered the 100 most-cited papers published in the last decade in each of the 21 scientific fields to which scientific endeavour is categorized by the Web of Science [4]. One may consider each field with its set of 100 most-cited papers as the equivalent of one ecosystem. Hence one can study how many “species” (journals) are represented and how many times each “species” is represented in the sample of n = 100. An ecosystem tends to have low species diversity when it is severe, i.e. few species can survive in it. Conversely, species diversity is large in areas that have mild environments and/or many distinct niches [5].

## Results

### Journals publishing most-cited papers

**Figure 1:**
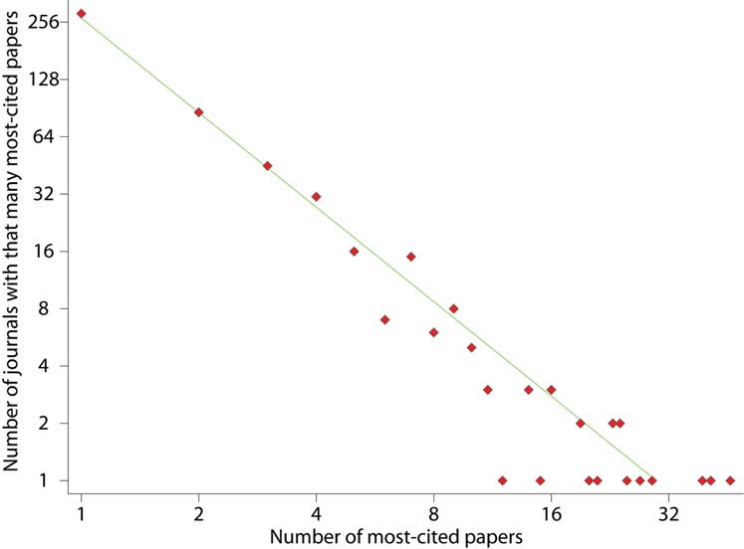
Number of journals that have published different numbers of papers that have been among the 100 most-cited in a scientific field in the last decade. R^2^ is 0.97 in regression weighted by the number of journals (R^2^ = 0.92 in unweighted regression, regression coefficients are similar). The graph does not show outlying *Science* and *Nature*.

The overall literature of most-cited papers is characteristic of low species diversity. Across all scientific fields combined, i.e. among 5,969 journals in the Journal Citation Reports of the Web of Knowledge, only 530 (9%) journals have published at least one paper that belongs to the 100 most-cited of a scientific field. Of those, 284 journals have published only a single such paper. The number of journals that have published a larger number of most-cited papers decreases exponentially with very good fit to a Lotka law. The power of the law is 1.6. The best-fit curve for the number of journals *y* that have published *n* top-cited papers is given by ln(*y*) = 5.6−1.6 x ln(*n*) with p<0.001 for both coefficients and R^2^ = 97% (Figure 1). *Nature* and *Science* have each published over 10% of the 2,100 examined most-cited papers (n = 231 and n = 227 respectively), followed by *Cell* (n = 46), *PNAS* (n = 41) and *Astrophysical Journal* (n = 39).

### Diversity in specific fields

Examination of each scientific field separately can give additional insights (Table 1). The journals that publish the largest number of most-cited papers are never the ones that publish the largest number of articles in their field, with the exception of the *Astrophysics Journal* in Space Science. With the exception of Engineering, General Social Sciences, and Economics/Business, two journals account for 14 to 46 of the 100 most-cited papers in each field, and 6 journals account for 53 to 94 of the 100 most-cited papers in each field. Thus in most scientific fields, there is limited diversity in the journals represented among those most influential publications, but this is not equally true across different disciplines.

**Table 1:**
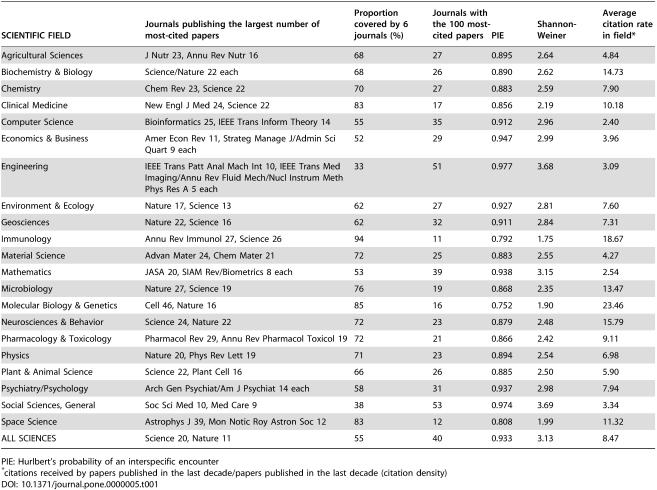
Diversity indices for journals publishing the 100 most-cited papers in 21 scientific fields

SCIENTIFIC FIELD	Journals publishing the largest number of most-cited papers	Proportion covered by 6 journals (%)	Journals with the 100 most-cited papers	PIE	Shannon- Weiner	Average citation rate in field*
Agricultural Sciences	J Nutr 23, Annu Rev Nutr 16	68	27	0.895	2.64	4.84
Biochemistry & Biology	Science/Nature 22 each	68	26	0.890	2.62	14.73
Chemistry	Chem Rev 23, Science 22	70	27	0.883	2.59	7.90
Clinical Medicine	New Engl J Med 24, Science 22	83	17	0.856	2.19	10.18
Computer Science	Bioinformatics 25, IEEE Trans Inform Theory 14	55	35	0.912	2.96	2.40
Economics & Business	Amer Econ Rev 11, Strateg Manage J/Admin Sci Quart 9 each	52	29	0.947	2.99	3.96
Engineering	IEEE Trans Patt Anal Mach Int 10, IEEE Trans Med Imaging/Annu Rev Fluid Mech/Nucl Instrum Meth Phys Res A 5 each	33	51	0.977	3.68	3.09
Environment & Ecology	Nature 17, Science 13	62	27	0.927	2.81	7.60
Geosciences	Nature 22, Science 16	62	32	0.911	2.84	7.31
Immunology	Annu Rev Immunol 27, Science 26	94	11	0.792	1.75	18.67
Material Science	Advan Mater 24, Chem Mater 21	72	25	0.883	2.55	4.27
Mathematics	JASA 20, SIAM Rev/Biometrics 8 each	53	39	0.938	3.15	2.54
Microbiology	Nature 27, Science 19	76	19	0.868	2.35	13.47
Molecular Biology & Genetics	Cell 46, Nature 16	85	16	0.752	1.90	23.46
Neurosciences & Behavior	Science 24, Nature 22	72	23	0.879	2.48	15.79
Pharmacology & Toxicology	Pharmacol Rev 29, Annu Rev Pharmacol Toxicol 19	72	21	0.866	2.42	9.11
Physics	Nature 20, Phys Rev Lett 19	71	23	0.894	2.54	6.98
Plant & Animal Science	Science 22, Plant Cell 16	66	26	0.885	2.50	5.90
Psychiatry/Psychology	Arch Gen Psychiat/Am J Psychiat 14 each	58	31	0.937	2.98	7.94
Social Sciences, General	Soc Sci Med 10, Med Care 9	38	53	0.974	3.69	3.34
Space Science	Astrophys J 39, Mon Notic Roy Astron Soc 12	83	12	0.808	1.99	11.32
ALL SCIENCES	Science 20, Nature 11	55	40	0.933	3.13	8.47

PIE: Hurlbert's probability of an interspecific encounter

* citations received by papers published in the last decade/papers published in the last decade (citation density)

Table 1 shows indices of alpha diversity (unevenness among represented journals) for these 21 ecosystems. The number of represented journals among the 100 most-cited papers, Hurlbert's probability of an interspecific encounter (PIE), and Shannon-Weiner indices have high correlation coefficients among themselves (0.86–0.97, p<0.001). There are considerable differences in these indices, however, across scientific disciplines. For example, Immunology as well as Molecular Biology & Genetics exhibit very high concentration in a few journals with PIE and Shannon-Weiner indices below 0.8 and below 2.0, respectively. Conversely, Engineering and General Social Sciences have no particular concentration and there is substantial diversity in the journals representing the top-cited papers; their PIE and Shannon-Weiner indices are above 0.97 and above 3.6, respectively.

### Correlates of species (journal) diversity

**Figure 2:**
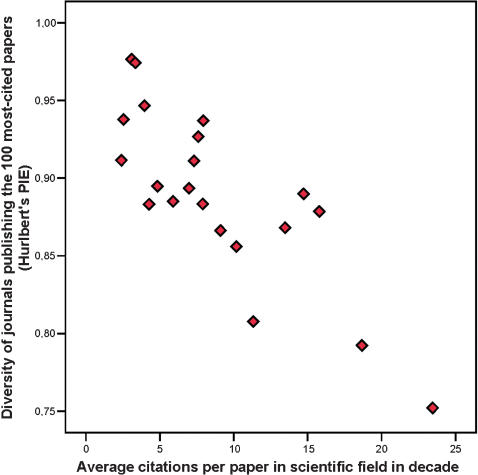
The diversity of journals publishing the 100 most-cited papers in a scientific field (here expressed by Hurlbert's PIE) is negatively related with the average number of citations per paper in the field (citation density).

Correlation analyses show that the diversity indices are not related to the number of published papers, the number of indexed journals, or the total citations received by journals in the field. For example, the Pearson correlations of these variables with Hurlbert's PIE are 0.02 (p = 0.94), 0.33 (p = 0.15), and −0.29 (p = 0.20), respectively. Conversely, diversity indices show strong negative correlations with the average number of citations received by each paper in the field, i.e. citation density (Figure 2): the Pearson correlation coefficients with number of journals, Hurlbert's PIE and Shannon-Weiner diversity index are −0.69 (p = 0.001), −0.82 (p<0.001) and −0.75 (p<0.001), respectively. Thus, with an increasing number of citations per paper in a scientific field, the preferential concentration of the most-cited papers in a few select journals becomes even more prominent.

Exclusion of the multidisciplinary journals from the calculations yields quite similar results with little attenuation in the correlation coefficients. Fields with higher citation density also concentrate their most-cited papers into fewer field-specific journals. The Pearson correlation coefficients are −0.64 (p = 0.002), −0.70 (p<0.001), and −0.67 (p = 0.001) between the average citation rate and the number of journals, Hurlbert's PIE and Shannon-Weiner index, respectively.

### Concentration in multidisciplinary journals

**Figure 3:**
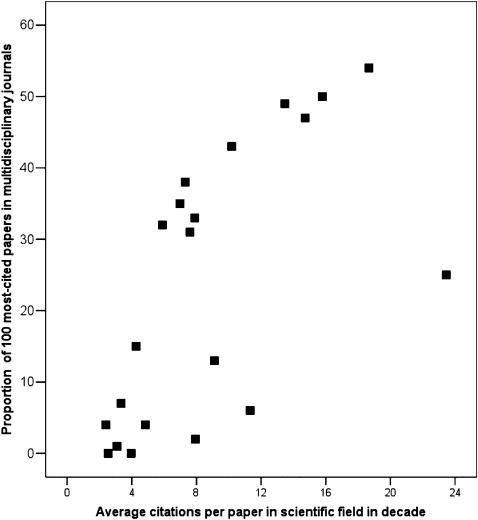
Relationship between the average number of citations in the field (citation density) and extent of concentration of the most-cited papers in multidisciplinary journals.

High impact multidisciplinary journals account for a quarter (24% [501/2100]) of the 2,100 analyzed most-cited articles across all 21 fields combined, and *Nature* and *Science* have the lion's share (458/501). However, there is a clear pattern that multidisciplinary journals concentrate most-cited papers preferentially from scientific fields with high average number of citations per paper (Pearson correlation coefficient 0.64, p = 0.002, Spearman correlation coefficient 0.70, p<0.001, Figure 3). Multidisciplinary journals account for 54 of the 100 most-cited papers in Immunology, 50 in Neurosciences & Behaviour, 49 in Microbiology, and 47 in Biochemistry and Biology. Conversely, they have published none of the 100 most-cited papers in Mathematics, or Economics & Business, and only 1 of the 100 most-cited papers in Engineering, and they also have little concentration (2–7 of the 100 most-cited papers in the field) in another 5 scientific fields (Psychiatry/Psychology, Agricultural Sciences, Computer Science, Space Science, General Social Sciences).

The relationship between citation density in the field and concentration of the most-cited papers in multidisciplinary journals is sigmoid, but there are 4 outliers to this pattern (Figure 3). For Molecular Biology and Genetics, Space Sciences, and Pharmacology and Toxicology, there is less concentration in multidisciplinary journals than what would be expected based on their high citation density; in these three fields, there are very strong specialty journals that attract a large number of most-cited papers (*Cell* n = 46, *Astrophysical Journal* n = 39, *Pharmacology Reviews* n = 29). This does not apply to the fourth outlier (Psychiatry/Psychology) where multidisciplinary journals also have no major presence (2/100 most-cited papers) despite modestly high citation density in the field.

### Original articles and reviews

A considerable number of the 100 most-cited articles in each field are apparently review articles. The distinction between original articles vs. reviews is often difficult. It may be more relevant in the biological/life sciences, where a distinctive feature may be the presentation of new data, but even this rule is not absolute. For example, *Ca – A Cancer Journal for Clinicians* publishes on an annual basis extremely highly-cited papers summarizing cancer statistics. While these are original data, they are practically annual reviews of the burden of disease due to cancer. The distinction between original articles and reviews is very impractical in the physical, mathematical, and social sciences, where several fully original papers are likely to deal with theoretical, conceptual, or mathematical constructs without specific empirical data.

Allowing for this caveat, table 2 shows the split between reviews and original articles among the most-cited papers in three life sciences, using conventional understanding of what would constitute a review in these fields. The concentration into very few journals was very prominent both for reviews and for original articles, when these were considered separately. Diversity indices showed similar or stronger concentration in each subgroup category compared with the overall analysis. However, the order of journals in the top ranks was different in each subgroup. For example, in Pharmacology and Toxicology, the two most common journals publishing most-cited papers were exclusively review journals (*Pharmacology Reviews* and *Annual Reviews of Pharmacology and Toxicology*); *PNAS* had published the largest number of highly-cited original articles, followed by *Molecular Pharmacology* and for both of these journals, all their most-cited papers were original articles. Review specialization of journals was less prominent for most-cited papers in some other fields, such as Clinical Medicine (Table 2), where the three journals with the highest number of most-cited papers published both original articles and reviews. However, even in this scientific field, some journals published only most-cited reviews (e.g. *Ca-A Cancer Journal*) and others published only most-cited original articles (e.g. *Lancet* and *PNAS*).

**Table 2:**
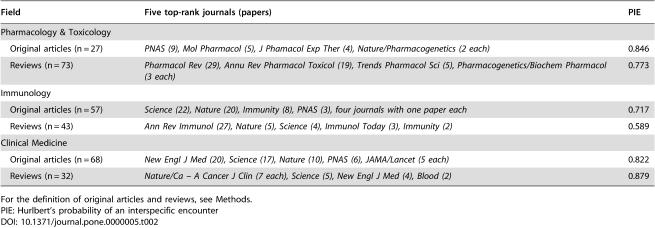
Ranking of journals publishing the highest number of most-cited original articles and reviews in three life sciences

Field	Five top-rank journals (papers)	PIE
Pharmacology & Toxicology		
Original articles (n = 27)	*PNAS (9), Mol Pharmacol (5), J Phamacol Exp Ther (4), Nature/Pharmacogenetics (2 each)*	0.846
Reviews (n = 73)	*Pharmacol Rev (29), Annu Rev Pharmacol Toxicol (19), Trends Pharmacol Sci (5), Pharmacogenetics/Biochem Pharmacol (3 each)*	0.773
Immunology		
Original articles (n = 57)	*Science (22), Nature (20), Immunity (8), PNAS (3), four journals with one paper each*	0.717
Reviews (n = 43)	*Ann Rev Immunol (27), Nature (5), Science (4), Immunol Today (3), Immunity (2)*	0.589
Clinical Medicine		
Original articles (n = 68)	*New Engl J Med (20), Science (17), Nature (10), PNAS (6), JAMA/Lancet (5 each)*	0.822
Reviews (n = 32)	*Nature/Ca – A Cancer J Clin (7 each), Science (5), New Engl J Med (4), Blood (2)*	0.879

For the definition of original articles and reviews, see Methods.

PIE: Hurlbert's probability of an interspecific encounter

### Extent of concentration of papers, citations, and most-cited papers

The concentration of the most-cited papers is even stronger than the already prominent concentration of the number of papers published and citations received. For example, in Molecular Biology and Genetics the 6 most prolific journals account for 17% of the papers published in the field, the 6 most-cited journals account for 35% of the citations received in the field, and 6 journals account for 85 of the 100 most-cited papers in the field. This increasing concentration to the leading journals (concentration in most-cited papers>citations received>papers published) is seen even for scientific fields with low citation densities. For example, in the General Social Sciences the 6 most prolific journals account for 4% of the papers published in the field, the 6 most-cited journals account for 12% of the citations received in the field, and 6 journals account for 38 of the 100 most-cited papers in the field.

**Figure 4:**
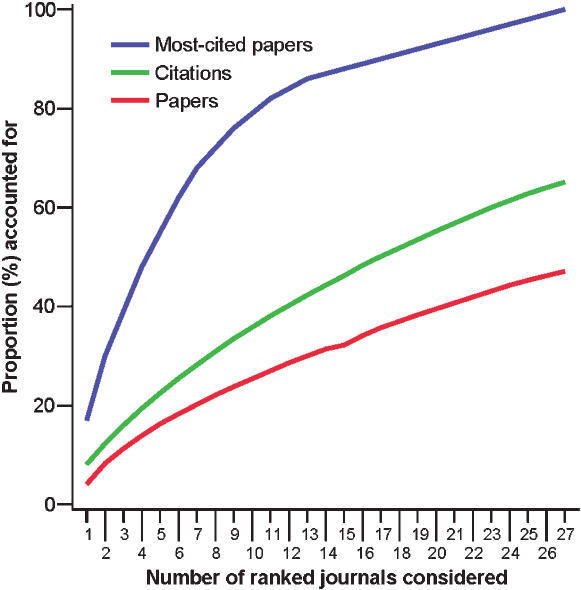
Proportion of papers, citations received and most-cited papers in the field of Environment & Ecology as a function of the number of journals considered. Journals are ranked according to number of papers, citations, and most-cited papers, respectively.

For example, for Environment & Ecology (a field with mid-range citation density), the 6 most-prolific journals account for 18% of the papers published in the field, the 6 most-cited journals account for 26% of the citations received, and 6 journals account for 62 of the 100 most-cited papers in the field. The strongest concentration of most-cited papers is also shown when cumulative proportion curves are plotted (Figure 4).

## Discussion

Influential scientific research is clearly highly concentrated, not only in terms of where it is produced [6]–[8], but also for where it is published. The most heavily cited scientific literature is compacted in a very small portion of the rapidly expanding base of scientific journal publications, although there are clear differences across scientific fields on the extent of this compaction. The compaction is generated by both multidisciplinary and field-specific specialized journals. However, existing multidisciplinary journals preferentially concentrate most-cited papers from scientific fields with high citation densities. The existing multidisciplinary journals have no or minimal share in the most influential scientific articles of 8 of the 21 examined scientific fields.

Despite an overwhelming plethora of available scientific journals, the optimal mode for scientific publishing remains controversial. In many fields, few journals cover most of the influential literature, regardless of the total number of journals circulating in the field and regardless of how many papers are published in the field. This may be interpreted as a strong tendency for centralization of the key literature, despite the large numbers of circulating periodicals. The existing high-quality, highly-competitive multidisciplinary journals (*Nature, Science*, and, to some extent, *PNAS*) contribute also to this centralization. However, their impact pertains primarily to fields where papers receive a lot of citations on average. For over a third of the examined scientific fields, the existing multidisciplinary journals currently have little or no presence in the high-impact literature. These fields include Mathematics, arguably the most rigorous science of all, where existing multidisciplinary journals have published none of the 100 most-cited papers in the last decade. Other sciences with very strong mathematical methodology and rigorous theoretical and applied methods such as Computer Science, Engineering, Space Science, Agricultural Sciences and practically all social sciences (General, Psychiatry/Psychology, Economics) are also not concentrated in these journals. The neglect for mathematics has been highlighted even by the demonstration of poorly applied routine mathematical/statistical methods in *Nature* [9]. The neglect of social sciences has been a recurring theme in scientific and funding circles [10], [11]. Multidisciplinary journals serve a critical role in avoiding fragmentation of science in times of over-specialization. Clearly there is a need also for multidisciplinary journals that would incorporate more of the mathematical, social, and applied sciences. For example, the launch of open-access PLoS ONE provides such an opportunity [12].

A previous study has shown that the journal of publication is the most important factor for a paper to receive citations - even more important than newsworthiness and quality that are also important for predicting future citation impact [13]. I should acknowledge that it is impractical, if not impossible, to find whether the citations received by the most-cited papers analyzed here were used appropriately or not by the citing authors. The motives for citing a paper are domain-specific; they may be affected by geographic location of authors and citers, number of authors, direction of results, and the length of a paper and potentially other factors as well [14], [15], but qualitatively they are almost chaotic to investigate in detail [16]–[18].

The concentration into a few journals was very prominent both for articles with original data and for reviews. However, the journals publishing most-cited reviews were often different from those publishing most-cited original articles. The high proportion of review articles among the most-cited literature in some scientific fields suggests that reviews deserve more attention as to the impact they have on scientific progress. It is difficult to tell whether citations to reviews are incidental casual references (e.g. something generic to cite in the start of the Introduction of a manuscript) or they also have a bearing on the scientific reasoning of the citing work. However, the same caveat applies also for most-cited articles with original data. Several empirical studies have documented the high citation rates of reviews and the fact that systematic reviews and meta-analyses performed with rigorous scientific methods for collecting, appraising and synthesizing information get more citations than non-systematic reviews [19]–[21]. In the physical, mathematical, and social sciences the distinction between review and original article is often impossible.

One should also acknowledge that there is no guarantee that the most-cited papers would even be “correct” or truly the “best” ones in the field. Controversy and refutation may also sometimes attract debate and citations [22]. As mentioned above, the social factors involved may be complex. This might include a need to cite those papers that everyone else is citing, yielding a citation concentration based on an attraction of the strongest. As the Nobel-prize novelist José Saramago has said, often people flock under the shadow of an opinion like under an umbrella [23]. In science, most-cited papers may be large tents where hundreds and thousands of scientists are flocking to. Nevertheless, citations are still a strong measure of impact and traffic of research among investigators. Of course, there is no perfect measure of impact. While other complementary measures of impact clearly need to be developed and appraised as well [24], citations also have many advantages [25] and currently enjoy relatively wide acceptance despite criticisms.

Allowing for these caveats, one may conclude that densely-cited scientific fields have the characteristics of severe, highly competitive ecosystems where few journals “survive” to attract the most influential papers. The number of journals operating in a field and the number of articles being published do not influence the extent of this concentration. Existing multidisciplinary journals target their efforts primarily in densely-cited fields. This is not so for scientific fields with lesser citation densities in which more journals survive in the top-rank ecosystems and where multidisciplinary journals currently have a minimal share.

## Methods

### Databases

The data of the Essential Science Indicators module of the Web of Knowledge, Thomson ISI that were used cover the period January 1, 1996 to March 1, 2006. Information on most-cited papers, number of journals, number of papers, average number of citations per paper, and total citations in each scientific field are derived from the Essential Science Indicators module. The categorization of journals by Thomson ISI is based on the citations received and given by each journal with clustering in 21 scientific fields and a miscellaneous/multidisciplinary category. Each journal is categorized in only one field, with the exception of a few journals such as *Science*, *Nature*, and *PNAS* for which each paper is allocated to a specific field based on the categorization of the journals that cite it.

### Alpha diversity indices

In order to evaluate the diversity of journals represented among the 100 most-cited papers in each field, I considered traditional indices of alpha diversity (unevenness among represented journals) for these journal ecosystems. These are the number of species in the ecosystem (number of journals), Hurlbert's probability of an interspecific encounter (PIE, the probability that a random sampling of two items from the 100 most-cited papers will yield the same species, i.e. papers in the same journal), and the Shannon-Weiner diversity index. PIE is given by
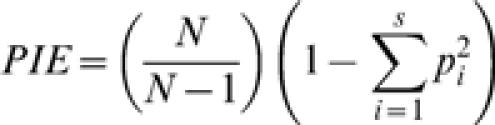
where N is the total sample size and *p_i_* is the proportion of the sample represented by species (journal) *i* with *i = 1…S*. The Shannon-Weiner index is given by
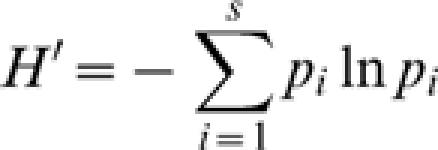
where *p_i_* is the proportion of the sample represented by species (journal) *i* with *i = 1…S*.

Calculations of diversity indices were performed in EcoSim version 7.72, Acquired Intelligence, Inc., Kelsey-Bear, 1997–2005.

The main analysis considered all journals in each field and a sensitivity analysis did not consider the multidisciplinary journals. Analyses for alpha diversity indices excluding the multidisciplinary journals used an appropriate rarefaction procedure so as to estimate the diversity indices based on the same number of papers in each scientific field. Alpha diversity indices cannot be compared across ecosystems, when the samples are of unequal size, because the indices depend on the sample size (with the exception of PIE). The number of papers published by multidisciplinary journals among the 100 most-cited ones ranged from 0 (Mathematics) to 54 (Immunology) and therefore all diversity indices are estimated here for abundance n = 46 (i.e. 100−54).

For the separate analyses of reviews and original articles, only PIE is presented that is not influenced by the different number of papers in these two categories. The category of “reviews” includes traditional reviews, systematic reviews, editorials, guidelines, consensus, classification and nomenclature papers.

Field-specific analyses also examined whether the extent of concentration into a few journals is similar for papers, citations, and most-cited papers. Journals were ranked according to number of papers published, citations received and most-cited papers, respectively. The proportion of papers, citations received and most-cited papers accounted was contrasted for the 6 top-ranked journals and was also shown with cumulative proportion curves.

### Other statistical analyses

Parametric (Pearson) and non-parametric (Spearman) correlation coefficients are reported, as appropriate. The power of the Lotka law is estimated with weighted least squares linear regression and the coefficient of determination (R^2^) is also provided. Spearman non-parametric coefficients are also reported when considered more appropriate. Statistical analyses were performed in SPSS version 13.0 (SPSS Inc., Chicago, Illinois) and p-values are two-tailed.
